# Simple graphical rules for assessing selection bias in general-population and selected-sample treatment effects

**DOI:** 10.1093/aje/kwae145

**Published:** 2024-06-20

**Authors:** Maya B Mathur, Ilya Shpitser

**Affiliations:** Quantitative Sciences Unit, Department of Medicine, School of Medicine, Stanford University, Palo Alto, CA 94304, United States; Department of Computer Science, Whiting School of Engineering, Johns Hopkins University, Baltimore, MD 21218, United States

**Keywords:** causal inference, collider stratification, effect-measure modification, generalizability, missing data, single-world intervention graphs

## Abstract

When analyzing a selected sample from a general population, selection bias can arise relative to the causal average treatment effect (ATE) for the general population, and also relative to the ATE for the selected sample itself. In this paper, we provide simple graphical rules that indicate (1) whether a selected-sample analysis will be unbiased for each ATE and (2) whether adjusting for certain covariates could eliminate selection bias. The rules can easily be checked in a standard single-world intervention graph. When the treatment could affect selection, a third estimand of potential scientific interest is the “net treatment difference”—namely the net change in outcomes that would occur for the selected sample if all members of the general population were treated versus not treated, including any effects of the treatment on which individuals are in the selected sample. We provide graphical rules for this estimand as well. We decompose bias in a selected-sample analysis relative to the general-population ATE into (1) “internal bias” relative to the net treatment difference and (2) “net-external bias,” a discrepancy between the net treatment difference and the general-population ATE. Each bias can be assessed unambiguously via a distinct graphical rule, providing new conceptual insight into the mechanisms by which certain causal structures produce selection bias.

## Introduction

Selection bias can arise as an inherent feature of data collection or could arise during analysis—for example, when handling missing data by conducting complete-case analysis or when conducting certain subset analyses.^1^ For example, a subset analysis can produce selection bias if one estimates the effects of obesity on mortality while restricting the analysis to participants who have a health condition (eg, heart disease) that may itself be affected by obesity.^2^ We use the term “selected-sample analysis” to refer to an analysis that is restricted to a nonrepresentative subset of the population of interest, regardless of whether this restriction arises from data collection, from analytical methods, or for any other reason. Depending on the mechanism of selection, an average treatment effect (ATE) estimated from a selected-sample analysis may be biased for the true, causal ATE in the general population prior to selection or even for the ATE in the selected sample itself.^1^^,^^3^^,^^4^

**Figure 1:**
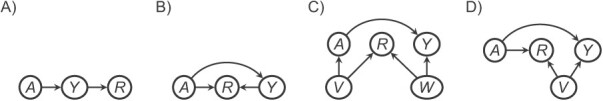
Directed acyclic graphs representing various causal structures that may produce selection bias. *A*, treatment; *Y*, outcome; *R*, indicator for selection into analysis; *V* and *W*, additional variables that may or may not be measured among the selected sample. Panel (A): *R* is affected by *A* (via *Y*) but is not a collider. Panels (B)-(C): *R* is a collider.

Figures 1A-1D depict four example structures in which selection bias could arise. Lu et al defined “internal validity” as “the case when the effect estimated from the analytic sample is equal to the true causal effect in the study sample”; they defined “external validity” as “the case when the true causal effect in the study sample is equal to the true causal effect in the target population”.^4^^(p.700)^ Others have given similar definitions.^3^^,^^5^ Lu et al suggested expressing selection bias arithmetically as the sum of “type 1” and “type 2” selection biases, as follows. “Type 1 selection bias” represents violations of internal validity (ie, a discrepancy between the estimate obtained conditional on selection versus the true causal ATE also conditional on selection), and “type 2 selection bias” represents violations of external validity (ie, a discrepancy between the true causal ATE conditional on selection versus the causal ATE not conditional on selection).^4^ Type 1 selection bias can arise, for example, if conditioning on the selection indicator in a causal graph induces a form of collider stratification bias, as in Figures 1B and 1C.^1^^,^^6^^,^^7^ (Accessible introductions to causal graphs and collider stratification are available.^6^^,^^8^ Briefly, a variable is a collider on a given path if the path has 2 arrowheads pointing into that variable—for example, $\to V\leftarrow$.) Type 2 selection bias can arise, for example, if selection into analysis is an effect-measure modifier, potentially because it is associated with unmeasured participant characteristics.^9^^,^^10^ (Accessible introductions to effect-measure modification are available.^8^^,^^10^^,^^11^)

Because each of these sources of bias can manifest in a diverse range of causal graphs,^3^^,^^4^ it would be helpful to establish simple graphical rules for when each bias may be present. In other words, just as one can assess whether confounding may be present in a given study by representing the causal structure via a graph and assessing whether there are unblocked backdoor paths between the exposure and the outcome,^12^ it would be helpful to have similar rules for each type of selection bias. Lu et al provided informal graphical rules for each source of bias, but as we will discuss and as others have noted,^13^ those rules do not seem to yield correct conclusions for certain causal structures.

Estimating the ATE or other estimands for the selected sample itself, rather than for the general population, may also be of scientific interest.^3^^,^^5^ In this context, an important challenge arises when the treatment affects selection into analysis: Namely, whether a given individual is in the selected sample depends on whether that individual is treated or not. That is, the “selected sample” is not a fixed group of individuals; the selected sample itself changes depending on who is treated. For this reason, to discuss causal effects or other estimands “among the selected sample” requires clarity about which individuals from the general population are actually being considered: (1) those who are in the selected sample we actually observe, based on the treatment those individuals actually received, or alternatively (2) those who *would be* in the selected sample if they were treated (or if they were not treated). We detail these issues with an example in the next section. Previous discussions of estimands that condition on the selection indicator have generally not specified which “selected sample” is in view.^4^^,^^5^^,^^14^ This can lead to substantial confusion when the treatment affects selection or any other conditioned variable.^15^ Similar challenges arise when decomposing selection bias in the ATE in the general population into type 1 and type 2 selection biases, because such decompositions themselves involve conditioning on the selection indicator.^4^

In this paper we will consider 3 estimands of interest, including the ATE in the general population and estimands pertaining to 2 different conceptions of “selected samples,” which will be defined precisely below. We will establish simple rules that can be checked in a causal graph to determine whether a selected-sample analysis, potentially conditional on additional covariates besides the selection indicator, will be unbiased for each quantity of interest. The graphical rules can be easily checked in a standard single-world intervention graph (SWIG)^15^^,^^16^; they do not require constructing modified graphs specifically designed for assessing selection bias.^17^^,^^18^ As we will illustrate through several examples, these rules will allow investigators to assess whether a selected-sample analysis will be unbiased for the estimand(s) of interest and to determine which covariates, if any, could be adjusted in order to eliminate selection bias.

We generalize Lu et al’s^4^ concepts of type 1 and type 2 selection bias to better accommodate the possibility that the treatment affects selection. To do so, we decompose selection bias into “internal bias” (a generalization of type 1 selection bias) and “net-external bias” (a generalization of type 2 selection bias). When the treatment does not affect selection, these are respectively equivalent to type 1 and type 2 selection bias: That is, internal bias arises from the discrepancy between the selected-sample estimate and the true causal ATE in the selected sample itself, and net-external bias arises from the discrepancy between the true causal ATEs in the selected sample and in the general population. We will discuss how these concepts generalize when the treatment does affect selection. Our proposed graphical rules will unambiguously indicate when each source of bias may be present, even if the treatment could affect selection. By mapping each source of selection bias onto a clear graphical rule, our results also provide conceptual insight into the specific mechanisms by which certain causal structures produce selection bias.

We focus primarily on the case in which there is no uncontrolled confounding in the general population, conditional on any covariates, other than the selection indicator, that have been controlled in analysis. In this case, any internal bias in the selected-sample analysis arises from conditioning on the selection indicator. However, most results we will present also generalize to the case in which there is also uncontrolled confounding, allowing a 1-step assessment of both sources of bias simultaneously.^19^^,^^20^ All results hold without making any assumptions about variable distributions or model link functions.

## Treatment-affected selection and the net treatment difference

**Figure 2:**
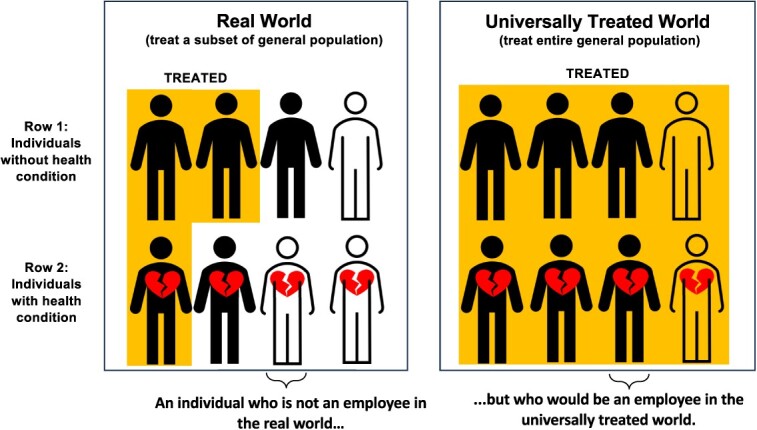
For the example of a workplace program designed to reduce sick days, a depiction of how the selected sample (current employees; left) could change if the program were implemented universally (right). The 8 people in each panel depict the general population (all individuals who have ever been employed by the company); the solid person icons indicate current employees, and the unfilled person icons indicate individuals who are no longer employees. Yellow boxes indicate individuals who are enrolled in the program in each world; in the real world, only a subset of the general population is treated. Red heart symbols indicate individuals with preexisting health conditions; one such individual is not an employee in the real world, but would be an employee if the program were universally in effect.

As noted in the Introduction, conceptual challenges arise when the treatment affects selection into analysis. In this case, in a hypothetical world in which the treatment was administered to all members of the general population, the individuals who would be in the selected sample are not necessarily the same individuals who were in the *factual* selected sample (ie, the selected sample that we actually observe and analyze in the real world). Consider an example represented by the graph in Figure 1D. Suppose a company hopes to reduce sick days (the outcome *Y*) among its employees by introducing a workplace program (the exposure *A*) to improve employees’ job control—for example, by allowing flexible hours and choice of tasks.^21^ The study is conducted among current employees (the selected sample, *R* = 1), who are a subset of all individuals who have ever been employees at this company (the general population). Employee characteristics such as having a chronic health condition (*V*) could affect both an employee’s frequency of taking sick days and their probability of staying with the company; thus, *R* could be an effect-measure modifier.^10^ Suppose the workplace program not only affects sick days ($A\to Y$) but also affects whether an employee chooses to stay with the company ($A\to R$). This means that, if the program were implemented universally, the company’s current employees (ie, the selected sample) could be different individuals from those who are currently employees in the real world. Figure 2 gives a simple example.

In this context, the causal ATE among the (factual) selected sample would represent the effect of the workplace program on sick days among individuals similar to the company’s current employees. However, since only a subset of the general population is in the program, the current employees might not resemble those who would be employees if the program were universally in effect. For example, current employees might be less likely to have a chronic health condition (Figure 2). The true causal ATE among the factual selected sample is an average of causal effects for each current employee, which does not include the causal effects for individuals who would only be current employees *if* the program had been universally in effect.

While this ATE might be of interest in some contexts, in this example the company might prefer to estimate a “net treatment difference” that captures not only causal effects of the treatment on specific individuals but also effects of the treatment on who is in the selected sample.^22^^,^^23^ That is, the company might be interested in the overall (ie, net) difference in employee sick days due to implementing the program, accounting for any effects of the program on which individuals are current employees in the first place. That is, if we compare a “universally treated world” in which all members of the general population are enrolled in the program with a “universally nontreated world” in which no members of the general population are enrolled, the net treatment difference is the average difference in sick days between individuals who would currently be employees in the universally treated world and those who would currently be employees in the universally nontreated world.^22^^,^^23^ In more general terms, the net treatment difference assesses the net change in outcomes that would occur for the selected sample if all members of the general population were treated versus not treated, including any effects of the treatment on which individuals are in the selected sample. Importantly, if the treatment affects selection, the net treatment difference does not, in general, represent a *causal* treatment effect because it does not compare potential outcomes for a fixed set of individuals.^23^ Again, this is because, for individuals like the one who is annotated in Figure 2, their membership in the selected sample depends on whether they are treated.

As noted in the Introduction, previous discussions of estimands that condition on the selection indicator have often not distinguished the factual selected sample from the counterfactual selected sample that would occur under a specific intervention on the treatment, and hence have not distinguished the causal ATE in the selected sample from the net treatment difference.^4^^,^^5^^,^^14^ It is important to note that even when the treatment affects selection, the net treatment difference may or may not be of scientific interest; this must be assessed in scientific context. Other estimands might additionally, or alternatively, be of interest in these contexts.^22^^‑^^26^ We focus on the net treatment difference for 2 reasons. First, we believe this estimand may be of scientific interest in some (though not all) contexts with treatment-affected selection.^22^^,^^23^ Second, and more importantly, we will show that decomposing sources of selection bias in a manner that refers to the net treatment difference provides conceptual insight into the mechanisms by which certain causal structures produce selection bias, and such a classification of selection biases can provide conceptual insight even when the net treatment difference is not itself of primary scientific interest.

## Setting and notation

Table 1 summarizes the terms and notation that will be introduced in this section and subsequently. We consider an analysis that is conducted among only a selected sample, denoted *R* = 1, and that involves a single treatment *A*, outcome *Y*, and a set of conditioned covariates *Z*. The variables may be of any type (eg, binary or continuous). The covariate set *Z* could be empty (ie, the analysis is unadjusted) or could include, for example, confounders or covariates that are controlled in an effort to eliminate selection bias.^3^^,^^17^^,^^27^ We assume that *A* does not affect any of the covariates in *Z*, which will hold if the covariates are measured before the treatment occurs. Thus, although we will consider a naive sample estimand that conditions on both *R* (due to selection into analysis) and *Z* (due to control in analysis), a key distinction is that *R* can be affected by *A*, but *Z* cannot. We make this assumption that *Z* does not contain treatment-affected covariates because controlling for such covariates is not necessary to eliminate confounding or selection bias,^12^^,^^14^ and doing so can introduce its own forms of bias, such as overadjustment bias.^8^^,^^28^^,^^29^

**Table 1:** Key terms and notation used in this paper

**Term**	**Definition**
Statistical terminology	
Estimand	Any population parameter that is to be estimated, such as an ATE
*R*-identified	Describes an estimand that can be unbiasedly estimated in a selected-sample analysis (ie, an estimand that is identified by ${\varDelta}_R$)
ATE	Average treatment effect
Net treatment difference	The net change in outcomes that would occur for the selected sample if all members of the general population were treated versus not treated, including any effects of the treatment on which individuals would be in the selected sample
Factual selected sample (*R* = 1)	The selected sample that we actually observe and analyze (ie, based on the treatments that individuals received in the real world)
Counterfactual selected sample ($R(a)=1$)	The individuals who would be in the selected sample if (perhaps counterfactually) they had received treatment $A=a$
Estimands of interest	
$\delta$	The causal ATE among the general population: $E\left[Y\left({a}_1\right)\,|\,Z=z\right]-E\left[Y\left({a}_0\right)\,|\,Z=z\right]$
${\delta}_R$	The causal ATE among the factual selected sample: $E\left[Y\left({a}_1\right)\,|\,R=1,Z=z\right]-E\left[Y\left({a}_0\right)\,|\,R=1,Z=z\right]$
${\delta}_{R(a)}$	The net treatment difference: $E\left[Y\left({a}_1\right)\,|\,R\left({a}_1\right)=1,Z=z\right]-E\left[Y\left({a}_0\right)\,|\,R\left({a}_0\right)=1,Z=z\right]$
Graphical terminology	
Collider	A variable on a path whose adjacent arrows both point into the variable, such as ${V}_j$ on the path ${V}_i\to{V}_j\leftarrow{V}_k$
Backdoor path between *A* and *Y*	A path of the form $A\leftarrow \cdots Y$
Noncausal path between *A* and *Y*	A nondirected path between *A* and *Y*, including backdoor paths
Confounding-sufficient set	A set of covariates which, if controlled in analysis, suffices to eliminate confounding in the general population

Abbreviation: ATE, average treatment effect.

To distinguish sources of internal bias that arise from “conventional” confounding in the general population (ie, the population prior to conditioning on *R*) versus those that arise only upon conditioning *R*, we will primarily consider sets *Z* that would suffice to control for confounding in a hypothetical analysis of the general population. To this end, if *Z* suffices to block all backdoor paths from *A* to *Y* when *R* is not conditioned, we will refer to *Z* as a “confounding-sufficient” set. (For any 2 variables *V* and *W*, a backdoor path from *V* to *W* is a nondirected path that contains an edge leading into *V*.^8^) All results will hold regardless of whether *Z* is confounding-sufficient, but as we will show, some results take a simplified form when *Z* is indeed confounding-sufficient.

For any given variable *V*, let *V*(*a*) denote the value that *V* would take under an intervention setting $A=a$. In particular, *R*(*a*) indicates whether a member of the general population would be in the selected sample if that individual were given treatment $A=a$. If *A* does not affect *R*, then *R*(*a*) is equal to the factual *R*; on the other hand, if *A* does affect *R*, then *R*(*a*) need not be equal to *R*. This statement is simply a formalization of the concepts presented heuristically in the above section entitled “Treatment-affected selection and the net treatment difference.” We make the standard assumptions of consistency (ie, for any variable *V*, we assume $V=V(a)$ if $A=a$) and positivity (ie, for a given conditioning set *z*, we assume $P\left(A=a\,|\,Z=z\right)>0$ for all values *z* of *Z* such that $P\left(Z=z\right)>0$).^8^

### Estimands of interest

The 3 estimands we will primarily consider, all conditional on *Z*, are as follows. For ease of notation, we express them all on the difference scale, but all results and graphical rules will apply regardless of the scale (eg, risk ratio, risk difference, etc) and regardless of the model link function. First, $\delta$ is the true, causal conditional ATE in the general population. For example, for treatment levels ${a}_1$ and ${a}_0$,


(1) \begin{equation*} \delta =E\left[Y\left({a}_1\right)\,|\,Z=z\right]-E\left[Y\left({a}_0\right)\,|\,Z=z\right]. \end{equation*}

Second, ${\delta}_R$ is the true, causal conditional ATE in the factual selected sample:


(2) \begin{equation*} {\delta}_R=E\left[Y\left({a}_1\right)\,|\,R=1,Z=z\right]-E\left[Y\left({a}_0\right)\,|\,R=1,Z=z\right]. \end{equation*}

Third, ${\delta}_{R(a)}$ is the conditional net treatment difference^22^:


(3) \begin{equation*} {\delta}_{R(a)}=E\left[Y\left({a}_1\right)\,|\,R\left({a}_1\right)=1,Z=z\right]-E\left[Y\left({a}_0\right)\,|\,R\left({a}_0\right)=1,Z=z\right]. \end{equation*}

Again, ${\delta}_{R(a)}$ represents the net change in outcomes that would occur for the selected sample if all members of the general population were treated versus not treated, including any effects of the treatment on which individuals are in the selected sample. A naive estimand obtained from a selected-sample analysis, which could be used to estimate any of the 3 estimands above, is


(4) \begin{equation*} {\varDelta}_R=E\big[Y\,|\,A={a}_1,R=1,Z=z\big)\big]-E\big[Y\,|\,A={a}_0,R=1,Z=z\big)\big]. \end{equation*}

We will provide graphical conditions under which ${\varDelta}_R$ is equal to $\delta$, ${\delta}_R$, ${\delta}_{R(a)}$, or several of these. We assume the analysis model for ${\varDelta}_R$ is correctly specified for the observed data; that is, any bias in ${\varDelta}_R$ relative to each of the estimands of interest reflects a faulty identification strategy, not misspecification of the model for the observed data. We will describe an estimand as “*R*-identified” if it is guaranteed to be equal to ${\varDelta}_R$ without requiring parametric assumptions or assumptions on the model link function; a more formal definition is given in Appendix S1 (Definition 1). That is, our proposed rules will indicate whether an estimand is *guaranteed* to be equal to ${\varDelta}_R$ without further assumptions. However, there will be cases in which the graphical conditions do not hold for a given causal structure but a given estimand is nevertheless equal to ${\varDelta}_R$ for a particular distribution or link function (eg, when estimating an odds ratio in the context of certain forms of outcome-affected selection^30^^,^^31^).

When *R*-identifiability is violated, this situation is more general than typical epidemiologic conceptions of selection bias, although multiple conceptions exist. Some authors have defined “selection bias” as any bias in ${\varDelta}_R$ relative to δ that arises due to conditioning on *R.*^4^^,^^14^ Others instead use “selection bias” specifically for bias that arises due to any noncausal path between *A* and *Y* that is unblocked because of conditioning on *R* or similarly, that arises from conditioning on a common effect of 2 other variables, namely (1) the treatment or a cause of the treatment and (2) the outcome or a cause of the outcome.^1^^,^^7^  *R*-identifiability differs from these definitions in 2 ways. First, whereas some (but certainly not all) epidemiologic definitions of “selection bias” specifically consider causal estimands, *R*-identifiability applies also to noncausal estimands such as ${\delta}_{R(a)}$. Second, violations of *R*-identifiability can arise not only when there is internal bias but also when there is effect-measure modification on any scale by *R*. Specifically, *R* will be an effect-measure modifier on at least one scale (eg, on the ratio scale or on the difference scale) if the distribution of potential outcomes *Y*(*a*) differs for the selected sample versus the general population. (These interpretations apply when *R* is not affected by *A*; we will later discuss how they generalize when instead *R* is affected by *A*.) As such, *R*-identifiability is similar to some epidemiologic definitions of “selection bias”^3^^,^^4^^,^^14^^,^^27^ but is more general than other definitions in which effect-measure modification alone (ie, without internal bias) is not conceptualized as selection bias.^1^^,^^7^

### **Decomposing the total bias relative to**  $\delta$

We will also consider the total additive bias of ${\varDelta}_R$ relative to $\delta$, namely $B={\varDelta}_R-\delta$. For mathematical tractability, we consider an additive bias decomposition regardless of the scale of ${\varDelta}_R$ itself. That is, let $B={B}_{\mathrm{int}}+{B}_{\mathrm{net}}$, where ${B}_{\mathrm{int}}={\varDelta}_R-{\delta}_{R(a)}$ and ${B}_{\mathrm{net}}={\delta}_{R(a)}-\delta$.^22^ Our ${B}_{\mathrm{int}}$ is internal bias in that it represents bias in the selected-sample estimand ${\varDelta}_R$ relative to the true net treatment difference, ${\delta}_{R(a)}$. Importantly, ${B}_{\mathrm{int}}$ is not necessarily equivalent to Lu et al’s “type 1 selection bias,” which is bias relative to ${\delta}_R$ rather than ${\delta}_{R(a)}$. If *A* does not affect *R*, the two definitions will coincide, but otherwise they generally will not coincide. If *Z* is confounding-sufficient, then ${B}_{\mathrm{int}}$ reflects bias that arises when conditioning on *R* creates an unblocked noncausal path between *A* and *Y*^6^—that is, a nondirected path that is unblocked conditional on $\left\{R,Z\right\}$ but is blocked conditional on *Z* alone. For example, in Figure 1D, the path $A\to R\leftarrow V\to Y$ is such a noncausal path if no covariates are adjusted (i.e., $Z=\{\}$). The graphical rules presented in the section entitled “Graphical rules for assessing selection bias in δ_*R*(*a*)_, δ*_R_*, and δ” formalize this concept.

Our ${B}_{\mathrm{net}}$ is a discrepancy between the net treatment difference ${\delta}_{R(a)}$ and $\delta$, whereas Lu et al’s “type 2 selection bias” is a discrepancy between ${\delta}_R$ and $\delta$. (Note that a discrepancy between two statistical parameters is not itself a bias; rather, the term "bias" here refers to the contribution of this discrepancy to selection bias.) Recalling that ${\delta}_{R(a)}$ is not a causal ATE, ${B}_{\mathrm{net}}$ is “net-external bias” that occurs when the distribution of potential outcomes *Y*(*a*) differs by *R*(*a*), in a sense formalized in Appendix S1. In simpler terms, this means that on average, how an individual’s outcome would respond to treatment differs across groups of individuals whose membership in the selected sample would be differently affected by treatment. If the treatment does not affect selection, this means that on average, how an individual would respond to treatment differs based on whether that individual is in the selected sample or not. As such, if the treatment does not affect selection, ${B}_{\mathrm{net}}$ essentially reflects effect-measure modification on any scale by *R* and is equivalent to Lu et al’s “type 2 selection bias.” However, again, if the treatment does affect selection, then ${B}_{\mathrm{net}}$ differs from Lu et al’s “type 2 selection bias.” We decompose by ${\delta}_{R(a)}$ rather than ${\delta}_R$ to improve conceptual clarity and scientific relevance when *R* may be affected by *A*, as in the example of the workplace program designed to reduce sick days. Additionally, as discussed below in the section entitled “Graphical rules for assessing selection bias in δ_*R*(*a*)_, δ*_R_*, and δ,” our proposed decomposition allows each source of bias to be assessed via a distinct graphical rule.

## Selection bias and graphical models

We now consider how causal graphs can help investigators determine which of the 3 estimands of interest can be estimated without bias in a selected-sample analysis; that is, which of them are *R*-identified. One could assess this by applying general, flexible results for causal inference using graphical models.^32^ However, our focus is on providing less general, simple graphical rules that are specific to our 3 estimands of interest and that are stated in terms of familiar graphical separation (d-separation) criteria. We hope these rules will be reasonably straightforward for applied epidemiologists to use.

Because a directed acyclic graph (DAG) does not depict the potential outcome *Y*(*a*), it is difficult to use rules of d-separation alone to directly assess whether *Y*(*a*) is statistically independent from factual variables, such as *A* and *R*. In simpler terms, a DAG depicts the values that variables actually take, not the values they would take if one were to intervene on the treatment. As others have discussed, this challenge can contribute to misleading assessments of selection bias.^13^^,^^15^^,^^33^ For example, Lu et al stated that “the mechanism of type 1 selection bias [ie, nonidentification of ${\delta}_R$] is that restricting to one (or more) level(s) of a collider (or descendant of a collider) opens a noncausal backdoor path between the exposure and the outcome.”^4^^(p.700)^ In Figure 1A, *R* is not a collider or a descendant of a collider, and there is not a backdoor path (ie, a path^6^^,^^8^^,^^12^^,^^34^ of the form $A\leftarrow \cdots Y$) unless one makes ad hoc modifications to the graph.^4^ Thus, Lu et al’s rule for type 1 selection bias^4^ seems not to hold here unless one makes ad hoc modifications, yet as Lu et al correctly note, ${\delta}_R$ is not *R*-identified. Whereas a direct application of Lu et al’s criterion to this graph would seem to indicate incorrectly that ${\delta}_R$ is *R*-identified, our proposed graphical rules will correctly and unambiguously establish that it is not. In Figure 1B, *R* is now an *A*–*Y* collider, and there is an unblocked path between *A* and *Y* conditional on *R*, but it is not a backdoor path per standard terminology.^6^^,^^8^^,^^12^^,^^34^ Under this standard interpretation, Lu et al’s criterion^4^ seems to indicate that ${\delta}_R$ should be *R*-identified, but this is not the case. If one interpreted “backdoor path” in Lu et al’s criterion to mean “backdoor paths or other noncausal paths,” then the criterion would yield the correct conclusion. Our proposed graphical rules will again establish the correct conclusion unambiguously.

Daniel et al^17^ established a generalized backdoor criterion for assessing whether $\delta$ is *R*-identified, whose application required an 8-step process of constructing multiple graphs with various edges added or removed. Others have provided perhaps simpler graphical rules for $\delta$,^35^ though these results also do not assess whether ${\delta}_{R(a)}$ and ${\delta}_R$ are additionally *R*-identified. In simpler terms, these results do not indicate whether a selected-sample analysis will be biased for ${\delta}_{R(a)}$ or for ${\delta}_R$. The graphical rules we will provide address the identification of all 3 estimands, and they require constructing only 1 standard graph. For example, the rules will allow the following immediate deductions. In Figures 1A and 1B, ${\delta}_{R(a)}$ is identified when no covariates are controlled, but ${\delta}_R$ and $\delta$ are not (in a sense formalized below). In Figure 1C, ${\delta}_{R(a)}$ and ${\delta}_R$ are identified conditional on *V*, but $\delta$ is not. However, all 3 are identified conditional on *W*. In Figure 1D, all 3 are identified conditional on *V*. The graphical rules we will provide apply for any causal structure, including those that are considerably more complicated than these examples.

In this paragraph, we formalize somewhat our discussion of graphs; readers seeking a general conceptual understanding could skip this material. We assume that an underlying, causal DAG can be specified for a general population, of which the selected sample is a strict subset. We consider the subgraph $G$ whose set of nodes is $\left\{A,Y,Z,R\right\}$, along with any common causes among the general population of all pairs of those variables. (More formally, the “common causes” of 2 variables *V* and *W* are those variables that are ancestors of both *V* and *W*. The ancestors of a given variable *V* are those variables that have directed paths into *V*.) Given this stipulation about common causes, we will generally assume that a nonparametric structural equation model with independent errors (NPSEM-IE) holds.^12^ This is the same model that is required for the identification of familiar causal estimands such as the natural direct and indirect effects in mediation analysis.^11^^,^^12^^,^^36^ However, we also provide a full set of graphical identification rules for more general causal models that need not have independent errors (Appendix S1, Theorem 1). Let $V$ be the variables in $G$ whose values are well-defined for every member of the general population.

Our graphical rules will use SWIGs, which directly depict relationships between variables in a hypothetical world in which an intervention has occurred.^15^^,^^16^^,^^37^ As such, SWIGs unify graphical and potential-outcome approaches to causality.^16^ Formal treatments of SWIGs in general contexts are given elsewhere^15^^,^^16^^,^^37^; here we summarize the basic principles in the context of a single intervention on *A*. We will specifically work with “single-world intervention templates” (SWITs), which are simply SWIGs that depict a general value of the treatment ($A=a$) rather than a specific numerical value (eg, *A* = 1).^16^ Like a SWIG, a SWIT contains not a single node for *A* but rather a node that has been split into 2 components: a random component representing the value of *A* prior to intervening on it and a fixed component representing its value after intervention, *a*. Any effects of other variables on *A* (ie, edges leading into *A* in the original DAG) are depicted in the SWIG by edges leading into the random component *A*, not the fixed component *a*. Heuristically, this is because any causal effects of other variables on *A* manifest only before an intervention setting *A* to a fixed value *a*; the intervened-upon value $A=a$ is not itself affected by other variables. Additionally, any effects that *A* has on other variables (ie, edges leading out of *A* in the DAG) are depicted in the SWIT by edges leading out of the fixed component *a*, not the random component *A*. Heuristically, after *A* has been set to *a*, it is this fixed value *a* that affects other variables, not the random value *A* that occurred prior to intervention. Additionally, other variables in the SWIT that are affected by *A* are relabeled as, for example, *V*(*a*).

Thus, a DAG ($G$) can be transformed into a SWIT corresponding to an intervention $A=a$, called $G(a)$, as follows^16^^,^^37^.

1. *Node-splitting*: Split the node for *A* into a random component labeled “*A*” and a fixed component labeled “*a*”. Any edges in the original DAG that lead into *A* are inherited by the random component in the SWIT, and any edges in the DAG that lead out of *A* are inherited by the fixed component. Thus, the random component *A* has no outgoing edges, and the fixed component *a* has no incoming edges.
2. *Minimal labeling*: For each other variable *V* that is affected by *A*, relabel it as *V*(*a*). For each other variable *W* that is not affected by *A*, retain its original labeling *W*; for these variables, $W=W(a)$ by definition.

For example, in Table 2, sections A-D show the SWITs corresponding respectively to the DAGs in Figure 1.

**Table 2:** For various single-world intervention templates, whether each condition (C2) is fulfilled (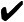) or violated (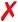) conditional on the specified set *Z*, and accordingly, whether each of 3 estimands is *R*-identified conditional on *Z* (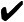) or not (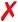), within the class of nonparametric structural equation models with independent errors

**Table section**	$\boldsymbol{\mathcal{G}(a)}$	***Z***	**Condition**		$\boldsymbol{{\delta}_{R(a)}}$	$\boldsymbol{{\delta}_R}$	$\boldsymbol{\delta}$
			C1	C2			
A	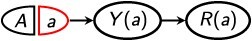	{}	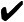	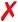	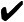	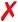	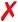
B	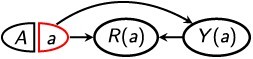	{}	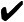	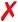	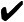	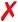	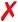
C	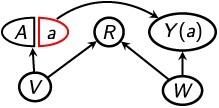	{}	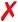	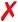	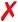	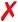	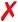
		*V*	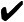	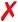	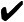	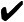	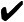
		*W*	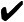	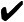	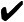	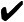	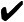
D	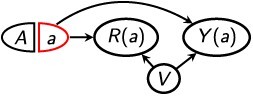	{}	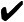	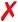	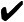	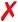	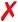
		*V*	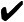	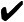	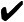	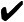	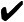
E	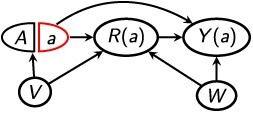	{}	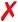	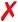	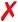	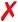	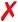
		*V*	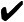	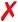	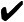	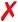	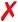
		*W*	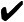	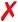	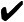	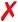	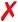
F	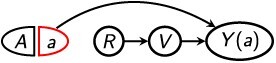	{}	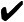	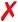	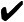	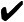	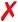
		*V*	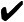	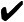	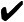	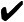	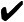
G	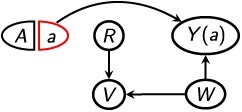	{}	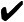	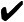	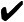	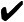	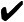
		*V*	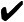	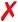	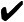	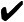	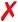
		*W*	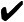	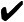	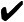	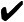	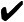

A SWIT can be used to assess whether variables are independent in the intervened-upon world simply using the usual rules of d-separation that apply to DAGs. The only additional stipulation is that in a SWIT, any path passing through the fixed node *a* is blocked. To briefly recap the usual rules of d-separation, a given path is “d-separated” or “blocked” by a set *D* if, for some set of variables $\{ {V}_i,{V}_j,{V}_k \}$: (1) the path contains a chain ${V}_i\to{V}_j \to{V}_k$ or a fork ${V}_i\leftarrow{V}_j\to{V}_k$, where ${V}_j\in D$; or (2) the path contains a collider ${V}_i\to{V}_j\leftarrow{V}_k$, where neither ${V}_j$ nor any of its descendants (ie, variables that are affected by ${V}_j$) is in *D.*^12^ A set *D* “d-separates” 2 variables *W* and *B* in a graph $M$ if and only if *D* blocks every path in $M$ between *W* and *B*,^12^ denoted ${\left(W\,\coprod \,B\,|\,D\right)}_M$. If ${\left(W\,\coprod \,B\,|\,D\right)}_M$, then *W* and *B* are statistically independent given *D*, denoted $\left(W\,\coprod \,B\,|\,D\right)$.

In any SWIT $M(a)$, if ${\left(W(a)\,\coprod \,B(a)\,|\,D(a)\right)}_{M(a)}$, then *W*(*a*) and *B*(*a*) are statistically independent given *D*(*a*)—that is, $\left(W(a)\,\coprod \,B(a)\,|\,D(a)\right)$.^37^ Because a SWIT depicts the random node-component *A*, one can directly assess conditional independence relationships between the factual treatment *A* and other variables’ counterfactual values in the intervened-upon world (which, for any variable that is not affected by *A*, will be equal to the variable’s factual value). Shpitser et al^37^ and Malinsky et al^38^ provided 3 rules, called the “potential outcome calculus” or “po-calculus,” from which all identification results that are valid for a given SWIT can be derived. Our results are derived using the po-calculus (Appendices S1 and S2), although applying the identification results in practice requires only familiarity with the standard rules of d-separation.

## **Graphical rules for assessing selection bias in**  ${\delta}_{R(a)}$**,**  ${\delta}_R$**, and**  $\delta$

We will use the following 2 graphical rules to establish when each of the 3 estimands is *R*-identified (ie, when a selected-sample analysis will be unbiased):


(C1) \begin{equation*} {\left(Y(a)\,\coprod \,A\,|\,R(a),Z\right)}_{G(a)} \end{equation*}

(C2) \begin{equation*} {\left(Y(a)\,\coprod \,R(a)\,|\,Z\right)}_{G(a)}. \end{equation*}

By referring to *Z* rather than *Z*(*a*), these conditions embed the assumption that none of the covariates in *Z* is affected by *A*, because otherwise *Z* would not appear in the SWIT at all^37^; instead, only *Z*(*a*) would appear (see “Minimal labeling” above). Condition 1 (C1) states that there is no path between *Y*(*a*) and *A* that is unblocked conditional on *R*(*a*) and *Z*, which would necessarily be a backdoor path.^37^ Condition 2 (C2) states that there is no path between *Y*(*a*) and *R*(*a*) that is unblocked conditional on *Z*. If such a path were present, it need not be a backdoor path. The latter condition can also be understood through the framework of principal stratification (Appendix S2).^23^ (Briefly, if C2 holds, this usually means that conditional on *Z*, the distribution of potential outcomes *Y*(*a*) is the same across principal strata that define whether each member of the general population would be in the selected sample if treated and if not treated.) We give examples of assessing the conditions for various SWITs in the “Examples” section.

Recall that the total additive bias of ${\varDelta}_R$ relative to $\delta$ can be decomposed as $B={B}_{\mathrm{int}}+{B}_{\mathrm{net}}$, where ${B}_{\mathrm{int}}={\varDelta}_R-{\delta}_{R(a)}$ and ${B}_{\mathrm{net}}={\delta}_{R(a)}-\delta$. We first discuss graphical rules that apply regardless of whether Z is confounding-sufficient, and then describe how the last rule takes a simplified form when *Z* is confounding-sufficient.

Proposition 1. If C1 holds, then ${\delta}_{R(a)}={\varDelta}_R$ and so *B*${}_{\mathrm{int}}$=0.

Proposition 2. If C1 holds and *R* is not affected by *A*, then ${\delta}_R={\delta}_{R(a)}={\varDelta}_R$ and so *B*${}_{\mathrm{int}}$=0.

Proposition 3. If C2 holds, then ${\delta}_{R(a)}=\delta$ and so *B*${}_{\mathrm{net}}$=0.

Proposition 4. If both C1 and C2 hold, then $\delta ={\delta}_R={\delta}_{R(a)}={\varDelta}_R$ and so $B={B}_{\mathrm{int}}={B}_{\mathrm{net}}=0$.

Thus, each source of bias can be assessed by a distinct graphical rule. That is, without assumptions on whether *R* is affected by *A*, C1 implies that *B*${}_{\mathrm{int}}$ = 0, and C2 implies that *B*${}_{\mathrm{net}}$ = 0. Decomposing *B* by *R*(*a*) rather than by *R*^4^^,^^5^ enables this 1:1 connection between graphical rules and sources of bias. As these results indicate, the issue of whether *R* is affected by *A* is relevant, in particular, to whether ${\delta}_R$ is *R*-identified. That is, if *R* is not affected by *A*, C1 alone is sufficient for ${\delta}_R$ to be *R*-identified. However, if instead *R* is affected by *A*, then C1 alone is *not* sufficient for ${\delta}_R$ to be *R*-identified; both C1 and C2 must hold. These results also highlight a key distinction between ${\delta}_R$ and the net treatment difference ${\delta}_{R(a)}$: If *R* is affected by *A*, and C1 holds but C2 is violated, then ${\delta}_{R(a)}$ is *R*-identified even though ${\delta}_R$ is not.

As shown in Appendix S2 (Lemma 2), if *Z* suffices to control for confounding in the general population, C2 implies C1. Therefore, Proposition 4 simplifies to:

Proposition 5. If C2 holds and *Z* is confounding-sufficient, then $\delta ={\delta}_R={\delta}_{R(a)}={\varDelta}_R$ and so $B={B}_{\mathrm{int}}={B}_{\mathrm{net}}=0$.

Note that Propositions 1-5 give graphical rules that are sufficient, but in some cases not necessary, for the given identification to hold (Appendix S3). Additionally, these results pertain to NPSEM-IE distributions. In Appendices S1 and S2 (Theorems 1 and 2), we consider a broader class of distributions, called “finest fully randomized causally interpretable structured tree graphs” (FFRCISTG), in which it is not assumed that the graph contains all common causes of variables in the graph.^39^ We give graphical rules for FFRCISTG distributions (Appendix S1), from which Propositions 1-5 follow as special cases.

## Examples

Table 2 gives a number of examples; the first 4 correspond to the DAGs in Figure 1. To provide intuition, we discuss several examples in applied contexts.

### Employee sick days

The example described above, regarding effects of a workplace program (*A*) on use of sick days (*Y*) among a company’s current employees (*R*), could conform to the SWIT in Table 2, section D. As discussed above, if the company’s goal is to assess the net difference in employee sick days due to implementing the program, accounting for the possibility that the program could affect which individuals would be current employees, the net treatment difference ${\delta}_{R(a)}$ may be of primary interest. Because C1 holds even without controlling for any covariates, the net treatment difference is identified. (Note that the path $a\to R(a)\leftarrow V\to Y(a)$ does not violate C1 because it involves the fixed node-component *a* rather than the random component *A*; this is because the path is not a backdoor path.) However, if the goal were instead to assess whether screening has a *causal* effect of reducing sick days among individuals similar to those who are current employees in the real world, then ${\delta}_R$ may be of primary interest. However, because *A* affects *R* and C2 is violated by the path $R(a)\leftarrow V \to Y(a)$, ${\delta}_R$ may not be identified if the analysis does not control for *V* (ie, having a preexisting health condition). Last, suppose the goal were to assess whether screening has, on average, a *causal* effect of preventing sick days among all individuals who have ever been employees, even those who no longer are. In this case, $\delta$ may be of interest. However, this estimand may not be identified because C2 is violated unless one controls for *V*. Since C1 holds, the internal bias *B*${}_{\mathrm{int}}$ = 0, so any bias in $\delta$ would be due to net external bias, ie, ${B}_{\mathrm{net}}\ne 0$.

### COVID-19 vaccination

The SWIT in Table 2, section C could correspond to a nonrandomized study on the effectiveness of COVID-19 vaccination (*A*) on COVID-19 infection (*Y*), in which data are available only for individuals who voluntarily obtained a COVID-19 test (*R*).^3^^,^^14^ Certain unmeasured characteristics, such as being generally risk-averse (*V*), could affect both vaccination status and the decision to obtain a COVID-19 test.^3^^,^^14^ However, we assume that vaccination itself does not affect the decision to obtain a test. Additionally, other unmeasured characteristics, such as exposure to a symptomatic individual (*W*), could affect both the decision to obtain a test and the risk of infection. If the goal is to estimate the causal effect of vaccination on infection among all individuals, $\delta$ may be of primary interest. If we do not condition on any covariates *Z*, then C1 is violated by the path $A\leftarrow V\to R\leftarrow W\to Y(a)$ and C2 is violated by the path $R\leftarrow W\to Y(a)$, so $\delta$ may not be identified. Specifically, both sources of bias, ${B}_{\mathrm{int}}$ and ${B}_{\mathrm{net}}$, may be present. However, if we condition on *W*, then both conditions (C1 and C2) hold, so $\delta$ is identified. Here, ${\delta}_{R(a)}={\delta}_R$ because vaccination does not itself affect the decision to obtain a test, and this represents the causal effect of vaccination on infection among only those individuals who choose to obtain tests. If we do not condition on any covariates, C1 is violated, so ${\delta}_R$ may not be identified. However, if we condition on *V*, *W*, or both, then the path $A\leftarrow V\to R\leftarrow W\to Y(a)$ is blocked, so C1 holds and ${\delta}_R$ is identified.

In a previously published study with this design, Bhattacharya et al^40^ assessed the association between being fully vaccinated against COVID-19 and becoming infected, as ascertained by reverse-transcriptase polymerase chain reaction. Participants were sampled from 2 sources: (1) individuals who visited a COVID-19 testing clinic in India (*n* = 583) and (2) hospitalized COVID-19 patients (*n* = 55). It appears that participants visited the testing clinic voluntarily, although details were not provided. Combining all participants, Bhattacharya et al^40^ found that being fully vaccinated was associated with greatly reduced odds of infection (unadjusted odds ratio = 0.17; 95% CI, 0.11-0.26). An analysis that adjusted for age and comorbid conditions yielded essentially the same estimate, which provides some reassurance if these variables constitute all the common causes (*W*) of choosing to obtain a test and becoming infected. If so, the authors’ adjusted odds ratio would be unbiased for the conditional ATE among all individuals ($\delta$) and among those who chose to obtain a test (${\delta}_R$). However, if there were other common causes, such as exposure to a symptomatic individual, there could be residual selection bias, in which case the authors’ adjusted odds ratio could be biased for both estimands.

### Smoking cessation

Finally, the SWIT in Table 2, section F could correspond to a longitudinal randomized study on the effects of financial incentives to quit smoking (*A*) on smoking cessation after 6 months (*Y*).^41^ We consider selection bias due to missing data in a complete-case analysis, such that the selected sample comprises participants who remain in the study for the 6 months of follow-up (ie, who do not drop out). In a previously published study with this design,^41^ all participants in both treatment groups received information about smoking-cessation programs. Thus, remaining in the study (*R* = 1) could affect participants’ ongoing access to educational information (*V*), which in turn could affect smoking cessation. In this example, C1 holds regardless of whether *V* is conditioned, and *R* is not affected by *A*, so ${\delta}_{R(a)}={\delta}_R$, and both are identified. However, C2 is violated unless *V* is conditioned, so in this case $\delta$ is not identified unless *V* is conditioned.

### Backdoor paths versus other noncausal paths

The SWITs in Table 2 also illustrate the distinct roles that backdoor paths, versus other noncausal paths, play in conditions C1 and C2. Recall that a noncausal path between *A* and *Y*(*a*) is any unblocked, nondirected path between *A* and *Y*(*a*), whereas a backdoor path is a specific type of noncausal path of the form $A\leftarrow \cdots Y(a)$. For example, the SWIT in Table 2, section C contains a backdoor path from *A* to *Y*(*a*) that is unblocked conditional on *R* alone. This path violates C1, and its subpath between *R* and *Y*(*a*) also violates C2. In contrast, the SWITs in sections B and D of Table 2 each contain a noncausal path between the fixed node-component *a* and *Y*(*a*). However, these are not backdoor paths, because the paths contain edges pointing out of *a*, rather than into *A*. Although these paths are unblocked conditional on *R*, they do not violate C1 because, as the SWIT depiction makes clear, edges that would point out of *A* in a DAG are instead inherited by the fixed part of the node *a* in the SWIT rather than the random part *A*. These noncausal paths do, however, contain subpaths between *R*(*a*) and *Y*(*a*) that violate C2 when only *R*(*a*) is conditioned.

### Additional results: Identification of a joint intervention on *A* and *R*

Proposition 4 indicates that if C1 holds but C2 is violated, $\delta$ may not be identified. We now consider an alternative estimand for the general population that nevertheless is *R*-identified in some such cases, and which may be of greater scientific interest in some contexts. Letting $Y\left(a,r\right)$ be the potential outcome under a joint intervention setting $\left(A=a,R=r\right)$, the alternative estimand is ${\delta}_r=E\left[Y\left({a}_1,1\right)\,|\,Z=z\right]-E\left[Y\left({a}_0,1\right)\,|\,Z=z\right]$. This represents, for the general population with $Z=z$, the average effect of intervening on *A* while also intervening to set *R* = 1 for all individuals in the general population.^42^

Because ${\delta}_r$ refers to an intervention on selection itself, this estimand represents a fundamentally different causal effect from the 3 we have focused on ($\delta$, ${\delta}_{R(a)}$, and ${\delta}_R$). As with those estimands, ${\delta}_r$ may or may not be of interest depending on the scientific context and the nature of the selection indicator. For example, ${\delta}_r$ may be particularly of interest in the selection arising from conducting a complete-case analysis. In this setting, the selected sample comprises participants who have no missing data, and intervening to set *R* = 1 represents “preventing” any data from being missing, which seems naturally of interest.^42^ On the other hand, in the aforementioned example of a workplace program designed to reduce sick days, ${\delta}_r$ represents the causal effect if the company were to not only implement the program but also prevent any employees from quitting. The latter is an intervention in its own right, and one which may have its own effects on sick days. However, this may not represent an intervention with any practical relevance, so ${\delta}_r$ may not be of interest in this study.

We will need the following graphical condition, which is a weakened version of C2:


(C2′) \begin{equation*} {\left(Y\left(a,r\right)\,\coprod \,R(a)\,|\,Z\right)}_{G\left(a,r\right)}, \end{equation*}

where $G\left(a,r\right)$ is a SWIT depicting interventions on both *A* and *R*. Such graphs are constructed using generalizations of the process described in the “Selection bias and graphical models” section (see Richardson and Robins^15^^,^^16^). By referring to *Z* rather than $Z\left(a,r\right)$, C2′ embeds not only our existing assumption that no variable in *Z* is affected by *A* but also a new assumption that no variable in *Z* is affected by *R*. We have the following analog to Proposition 4.

Proposition 6. *Identification conditions for*  ${\delta}_r$: If C1 and C2′ hold, then ${\delta}_r$ is *R*-identified.

**Figure 3:**
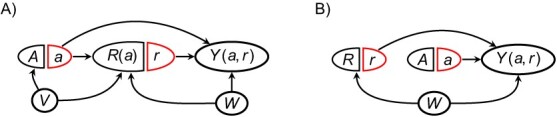
Single-world intervention templates, $G\left(a,r\right)$, depicting a joint intervention on *A* and *R*.

C2′ is weaker than C2 in that C2 implies C2′ but not vice versa. This is because if, in a DAG, there is a directed path from *R* to *Y* that is not blocked by *Z*, then C2 regarding $G(a)$ will be violated, but C2′ regarding $G\left(a,r\right)$ may still be fulfilled. For example, Figure 3A depicts the same mediation structure as in Table 2, section E, in which C1 holds conditional on *W*. Here, C2 is violated even conditional on *W*, but C2′ holds conditional on *W*. Therefore, ${\delta}_r$ is identified conditional on $Z=W$, even though $\delta$ may not be. Because *R* is a mediator in this structure, ${\delta}_r$ is equivalent to a controlled direct effect evaluated at *r* = 1.^11^^,^^12^^,^^36^ It is well-known that the controlled direct effect is identified if the statistical independence conditions $\left(Y\left(a,r\right)\,\coprod \,A\,|\,Z\right)$ and $\left(Y\left(a,r\right)\,\coprod \,R\,|\,\left\{A,Z\right\}\right)$ both hold.^11^^,^^12^^,^^36^ The graphical conditions C1 and C2′ are sufficient for the statistical conditions to hold,^36^ but the latter assumption also directly encodes an assumption that *R* is not affected by *Y*. This is generally assumed to hold in mediation analysis, but it need not hold in the context of selection bias.^11^^,^^12^^,^^36^ Note that C2′ can hold even if *R* is not a mediator, and even if *R* temporally precedes *A*. For example, in Figure 3B, C1 and C2′ both hold conditional on $Z=W$, but C2 is violated. Therefore, again, ${\delta}_r$ is identified conditional on $Z=W$, even though $\delta$ may not be.

## Discussion

We have primarily considered 3 estimands that may be of scientific interest when conducting a selected-sample analysis, namely the conditional ATE in the general population ($\delta$), the conditional ATE in the selected sample (${\delta}_R$), and the net treatment difference (${\delta}_{R(a)}$). We additionally considered a fourth estimand, representing the effect of intervening on the treatment while also intervening to set *R* = 1 for all members of the general population. Any or all of these estimands may be of inherent scientific interest, depending on context. To help demystify when a selected-sample analysis, potentially adjusted for covariates, will be unbiased for each of these estimands, we have provided simple graphical rules that are straightforward to check in a SWIT. These rules allow investigators to assess whether a selected-sample analysis will be unbiased for the estimand(s) of interest and to determine which covariates, if any, could be adjusted in order to eliminate selection bias.

Considering the net treatment difference ${\delta}_{R(a)}$ enabled a 2-way decomposition of bias relative to the causal ATE in the general population into (1) internal bias relative to the net treatment difference ${\delta}_{R(a)}$ and (2) net-external bias, namely a discrepancy between the net treatment difference ${\delta}_{R(a)}$ and the general-population ATE $\delta$. Net-external bias essentially reflects effect-measure modification on any scale by characteristics that are associated with whether an individual is in the selected sample if treated and if not treated. In contrast to previous decompositions,^4^ our proposed decomposition allows each source of bias to be assessed unambiguously in a graph, even when the treatment affects selection.

We considered estimands that are conditional on an arbitrary set of covariates, *Z*, that are not affected by the treatment, and we showed that the graphical rules simplify when *Z* suffices to control for confounding in the general population. Although conditioning on suspected confounders is common practice in epidemiology, less common is attempting to control for variables that would reduce or eliminate selection bias. Depending on the causal structure, it may or may not be possible to do so. Our proposed graphical rules could help assess whether the causal structure is such that covariate adjustment can eliminate selection bias in a given estimand of interest and if so, which specific covariates suffice. However, doing so requires knowledge of the full causal structure, which may not always be available. Other recent results apply if the causal structure is not fully known but is such that appropriate covariate adjustment can eliminate selection bias in the ATE for the general population.^27^ In this case, adjusting for all common causes of the outcome and selection, excluding the treatment and variables affected by the treatment, will eliminate selection bias in the ATE for the general population.^27^ In summary, we hope that our proposed graphical rules, which can be straightforwardly assessed in a standard SWIT, will help clarify sources of selection bias and inform the conduct and interpretation of analyses that condition on a selection indicator.

## Supplementary Material

Web_Material_kwae145

## Data Availability

No original data were used.
