# Supplementary material for: Simple graphical rules for assessing selection bias in general-population and selected-sample treatment effects
Source: Am J Epidemiol. 2024 Jun 20;194(1):267–77. doi: 10.1093/aje/kwae145 (PMC11735978; doi:10.1093/aje/kwae145)
Supplement: Web_Material_kwae145 [file web_material_kwae145.zip › kwae145 Mathur Supplementary Material.pdf]

Supplementary Material

Simple graphical rules for assessing selection bias in general-population and selected-sample treatment effects

Maya B. Mathur and Ilya Shpitser

Contents

Appendix S1: Proofs of main results **2**

Appendix S2: Proofs of additional results **4**

Special case for a confounding-sufficient Z 4

Identification of a joint intervention on A and R 5

A principal stratification view of condition (C2) 5

Appendix S3: Numerical example of identification in class $\mathcal{N}$ but not $\mathcal{F}$ **7**

Appendix S1: Proofs of main results

Recall that R is a singleton indicator for selection into analysis, A is a singleton treatment for which the contrast of interest is between levels a_0 and a_1, Y is a singleton outcome, and Z is a set of conditioned covariates, such that no member of Z is affected by A. For a given variable V, let $De(V)$ be its set of descendants and $An(V)$ its set of ancestors. We assume that an underlying, causal DAG can be specified for a general population, of which the selected sample is a strict subset. We consider the subgraph $\mathcal{G}$ whose set of nodes is $\{A, Y, Z, R\}$, and any shared ancestors of pairs of those variables. The SWIT $\mathcal{G}(a)$ corresponds to an intervention on A, and when discussing graphical conditions, we refer to $\mathcal{G}(a)$ unless otherwise stated. Whereas the main text focuses on identification within the class of NPSEM-IEs, we prove results for the broader class of FFRCISTG distributions and establish the results in the main text as special cases.

The main text considers whether any of the estimands $\delta_{R(a)}$, δ_R, or δ is identified by Δ_R. We first formalize the definition of R-identifiability, which will be used in stating Theorem 1.

Definition 1 (R-identifiability)**.** *For a given graph $\mathcal{M}$, let $\mathcal{D}$ be a given class of compatible distributions. For a given conditioning event S, the corresponding estimand $p(Y(a_1) \mid S) - p(Y(a_0) \mid S)$, is said to be R-identified for class $\mathcal{D}$ if, for any distribution in $\mathcal{D}$, we have:*

$$p\left(Y(a) \mid S\right) = p\left(Y \mid A = a, R = 1, Z = z\right),\ a \in \{a_0, a_1\}$$

Thus, if R-identifiability holds for $S = \{Z = z\}$, then $\delta = \Delta_R$ is guaranteed to hold without parametric assumptions, which is the sense in which we describe "R-identification" in the main text for simplicity. Similarly, if R-identifiability holds for $S = \{R(a) = 1, Z = z\}$, i.e., $p\left(Y(a) \mid R(a) = 1, Z = z\right) = p\left(Y \mid A = a, R = 1, Z = z\right)$, then $\delta_{R(a)} = \Delta_R$; and if R-identifiability holds for $S = \{R = 1, Z = z\}$ then $\delta_R = \Delta_R$.

Let $\mathcal{F}$ be the class of FFRCISTGs that are compatible with $\mathcal{G}$, and let $\mathcal{N} \subset \mathcal{F}$ be the class of NPSEM-IEs that are compatible with $\mathcal{G}$. We first prove sufficient identification criteria for the class $\mathcal{F}$ (Theorem 1). Recall the following graphical identification criteria:

$$\left(Y(a) \perp\!\!\!\perp A \mid R(a), Z\right)_{\mathcal{G}(a)} \tag{C1}$$

$$\left(Y(a) \perp\!\!\!\perp R(a) \mid Z\right)_{\mathcal{G}(a)} \tag{C2}$$

We can now establish the main identification results.

Theorem 1 (Necessary and sufficient identification conditions for FFRCISTGs)**.** *For the class of models $\mathcal{F}$: (i) $\delta_{R(a)}$ is R-identified if (C1) holds; (ii) δ_R is R-identified if (C1) holds and $R \notin De(A)$; and (iii) δ is R-identified if (C1) and (C2) hold.*

Proof. By (C1), applying Rule 2 of po-calculus [1] yields claim (i):

$$p(Y(a) \mid R(a) = 1, Z = z) = p(Y \mid A = a, R = 1, Z = z) \tag{S1}$$

If we additionally assume that $R \notin De(A)$, the left-hand side is $p(Y(a) \mid R = 1, Z = z)$, so claim (ii) holds. Considering claim (iii), note that condition (C2) alone has the following implication from Rule 1 of po-calculus:

$$p(Y(a) \mid R(a) = 1, Z = z) = p(Y(a) \mid Z = z) \tag{S2}$$

If we assume both (C1) and (C2) (without assuming $R \notin De(A)$), then combining Eqs. (S1) and (S2) establishes claim (iii). □

The R-identification results in Propositions 1 and 2 in the main text consider the class of NPSEM-IE distributions, i.e., $\mathcal{N} \subset \mathcal{F}$, so those propositions follow immediately from claims (i) and (iii) in Theorem 1. Proposition 3, which stated that condition (C2) implies $\delta_{R(a)} = \delta$, is established in Eq. (S2) in Theorem 1.[a] Proposition 4 also follows from Theorem 1 along with the following result that is specific to the class $\mathcal{N}$.

Lemma 1 (Sufficient identification conditions for NPSEM-IEs)**.** *For the class of models $\mathcal{N}$, δ_R is R-identified if conditions (C1) and (C2) hold.*

Proof. Under independent errors, condition (C2) alone implies that, for every $\{a, a'\}$ in the support of A, we have $p(Y(a) \mid R(a'), Z = z) = p(Y(a) \mid Z = z)$. Therefore, considering the factual R, we have:

$$p(Y(a) \mid R = 1, Z = z) = p(Y(a) \mid Z = z) \tag{S3}$$

[a] That result isn't its own numbered claim in the Theorem because it's not an R-identification result.

As in Theorem 1, conditions (C1) and (C2) imply that Eqs. (S1) and (S2) hold. Combining these with Eq. (S3) yields the desired result:

$$p(Y(a) \mid R = 1, Z = z) = p(Y \mid A = a, R = 1, Z = z)$$

$\square$

Appendix S2: Proofs of additional results

Special case for a confounding-sufficient Z

The following result formed part of Mathur et al.'s proof of their Lemma 3 [2]; we provide the relevant result and its proof here for completeness.

Lemma 2. *Suppose that Z is confounding-sufficient, i.e., $(Y(a) \amalg A \mid Z)_{\mathcal{G}(a)}$. Then condition (C2) implies condition (C1). That is, if $(Y(a) \amalg R(a) \mid Z)_{\mathcal{G}(a)}$, then $(Y(a) \amalg A \mid R(a), Z)_{\mathcal{G}(a)}$.*

Proof. Since $(Y(a) \amalg A \mid Z)_{\mathcal{G}(a)}$ and $(Y(a) \amalg R(a) \mid Z)_{\mathcal{G}(a)}$, we have $(Y(a) \amalg \{A, R(a)\} \mid Z)_{\mathcal{G}(a)}$ by the compositionality of d-separation in SWITs [3]. By the graphoid of weak union,[b] this implies $(Y(a) \amalg A \mid R(a), Z)_{\mathcal{G}(a)}$. $\square$

Combining this lemma with Lemma 1 and Theorem 1 yields the following two results.

Theorem 2 (Sufficient identification conditions for FFRCISTG with confounding-sufficient Z.)**.** *Suppose that Z is confounding-sufficient. Then, for the class of models $\mathcal{F}$: (i) $\delta_{R(a)}$ and δ are R-identified if (C2) holds; (ii) $\delta_{R(a)}$, δ_R, and δ are R-identified if (C2) holds and $R \notin De(A)$.*[c]

Proposition 5 (Sufficient identification conditions for NPSEM-IEs with confounding-sufficient Z.)**.** *Suppose that Z is confounding-sufficient. Then, for the class of models $\mathcal{N}$: $\delta_{R(a)}$, δ_R, and δ are R-identified if (C2) holds.*

[b]Graphoids are a set of statements about conditional independence that hold for graphical models (Pearl [4], Section 1.1.5). The graphoid of weak union states that for any four sets W, B, D, and F in a graph in a graph $\mathcal{M}$, if $(W \amalg \{B, D\} \mid F)_{\mathcal{M}}$, then $(W \amalg B \mid \{D, F\})_{\mathcal{M}}$ (Pearl [4], Section 1.1.5).

[c]Not "only if" anymore because the $C2 \Rightarrow C1$ result is one-directional.

Identification of a joint intervention on A and R

Recall the following weakened version of condition (C2):

$$(Y(a,r) \perp\!\!\!\perp R(a) \mid Z)_{\mathcal{G}(a,r)} \tag{C2'}$$

Proposition 6 (Identification conditions for δ_r)**.** *For the class of models $\mathcal{F}$, δ_r is R-identified if (C1) and (C2') hold.*

Proof. Condition (C1) again implies:

$$p(Y(a) \mid R(a) = 1, Z = z) = p(Y \mid A = a, R = 1, Z = z)$$

By (C2') and Rule 2, the left-hand side is equal to $p(Y(a, r = 1) \mid Z = z)$. □

A principal stratification view of condition (C2)

For a binary treatment, condition (C2) can be recast in the framework of principal stratification to provide additional intuition. For a binary treatment $A \in \{0, 1\}$, principal strata are fixed baseline characteristics that categorize members of the target population by their values of pairs of counterfactual values of a given variable, in this case $(R(0), R(1))$ [5]. Define $S = s_{ij}$ as the principal stratum comprising exactly those members of the target population for whom $(R(0) = i, R(1) = j)$. That is, the two levels of A and of R define four principal strata, within which all members have the same values of $(R(0), R(1))$. For example, the stratum s_{01} comprises individuals who would not be in the selected sample under an intervention setting $A = 0$ (i.e., $R(0) = 0$), but would be in the selected sample under an intervention setting $A = 1$ (i.e., $R(1) = 1$). The stratum s_{11} comprises individuals who would be in the selected sample regardless of which treatment they are, perhaps counterfactually, assigned. Even for members of the factual selected sample $R = 1$, the principal stratum for a given individual is not known without additional assumptions. This is because, for a given member of the factual selected sample, we observe that $R(A) = 1$ (i.e., we know that the individual is in the selected sample under their factual treatment assignment) but do not observe $R(1 - A)$ (i.e., we do not know whether the individual would have been in the selected sample had they received the treatment that they did not factually receive). By definition, S is not affected by A even if R itself is affected by A [5].

We define "stratum exchangeability" as exchangeability of the potential outcomes $Y(a)$ across the four principal strata, conditional on Z. That is, for a given distribution, stratum exchangeability is defined as the following condition being fulfilled for all $a \in \{0,1\}$ and $\{i,j,k,m\} \in \{0,1\}^4$:

$$p(Y(a) \mid S = s_{ij}, Z = z) = p(Y(a) \mid S = s_{km}, Z = z) \tag{C3}$$

Stated otherwise, this condition states that the distribution of potential outcomes $Y(a)$ is the same across principal strata, conditional on Z. As a special case, if $R \notin De(A)$, condition (C3) simplifies to $p(Y(a) \mid S = s_{00}, Z = z) = p(Y(a) \mid S = s_{11}, Z = z)$ for $a \in \{0,1\}$ because $p_{01} = p_{10} = 0$.

The next result states that for distributions that are faithful to $\mathcal{G}(a)$, if stratum exchangeability holds, then $\mathcal{G}(a)$ must fulfill condition (C2). A version of the converse also holds: if $\mathcal{G}(a)$ fulfills condition (C2), then this "usually" implies that stratum exchangeability holds. The latter is not a guaranteed implication; it holds when considering distributions that are not fine-tuned in the sense of implying certain perfect cancellations in probabilities, discussed below.

Proposition 7. *Suppose that $A \in \{0,1\}$. Define the principal strata S such that $S = s_{ij}$ exactly when $\mathbb{1}\{R(0) = i, R(1) = j\} = 1$ for $\{i,j\} \in \{0,1\}^2$. Let $M \in \mathcal{F}$ be an arbitrary distribution that is compatible with $\mathcal{G}$ and is faithful to $\mathcal{G}(a)$. Then:*

(i) Suppose that M fulfills stratum exchangeability (condition (C3)). Then $\mathcal{G}(a)$ fulfills condition (C2), i.e., $(Y(a) \perp\!\!\!\perp R(a) \mid Z)_{\mathcal{G}(a)}$.

(ii) Suppose $\mathcal{G}(a)$ fulfills condition (C2). If no assumptions are made on the stratum probabilities $P(S = s_{ij} \mid Z = z)$ other than simply $\sum_{ij} P(S = s_{ij} \mid Z = z) = 1$, then M must fulfill condition (C3).

Proof. By faithfulness, condition (C2) is equivalent to having the following statistical independence in M [4]:[d]

$$p(Y(a) \mid R(a) = 0, Z = z) = p(Y(a) \mid R(a) = 1, Z = z), \; a \in \{0,1\}$$

[d] Pearl Thm 1.2.5 for bidirectionality

Considering the intervention $a = 0$, this is equivalent to:

$$p(Y(0) \mid S \in \{s_{00}, s_{01}\}, Z = z) = p(Y(0) \mid S \in \{s_{11}, s_{10}\}, Z = z) \tag{S4}$$

Let $p_{ij} = P(S = s_{ij} \mid Z = z)$ and $p_{1\bullet} = P(S \in \{s_{11}, s_{10}\} \mid Z = z) = P(R(0) = 1 \mid Z = z)$. Noting that $p_{01} = 1 - p_{1\bullet} - p_{00}$ and $p_{10} = p_{1\bullet} - p_{11}$, Eq. (S4) is equivalent to:

$$\begin{aligned} &\frac{p_{00}}{1 - p_{1\bullet}} \cdot p(Y(0) \mid S = s_{00}, Z = z) + \frac{1 - p_{1\bullet} - p_{00}}{1 - p_{1\bullet}} \cdot p(Y(0) \mid S = s_{01}, Z = z) \\ &= \frac{p_{11}}{p_{1\bullet}} \cdot p(Y(0) \mid S = s_{11}, Z = z) + \frac{p_{1\bullet} - p_{11}}{p_{1\bullet}} \cdot p(Y(0) \mid S = s_{10}, Z = z) \end{aligned} \tag{S5}$$

Thus, condition (C2) is equivalent to Eq. (S5). Note that the probabilities p_{00}, p_{01}, and $p_{1\bullet}$ are not, in general, deterministically related.

To establish claim (i), we will show that condition (C3) implies Eq. (S5). Let $q = p(Y(0) \mid S = s_{ij}, Z = z)$; by condition (C3), this is constant in $\{i, j\}$. Thus, the left-hand side of Eq. (S5) is $\frac{p_{00}+1-p_{1\bullet}-p_{00}}{1-p_{1\bullet}} q = q$, and the right-hand side is $\frac{p_{11}+p_{1\bullet}-p_{11}}{p_{1\bullet}} q = q$, so Eq. (S5) holds. This establishes claim (i).

To establish claim (ii), we will show that Eq. (S5) implies condition (C3), unless M is fine-tuned in that its stratum membership probabilities lead to certain perfect arithmetic cancellations. Arguing by contradiction, suppose Eq. (S5) holds but that condition (C3) is violated. That is, for some $\{i, j, k, m\} \in \{0, 1\}^4$, we have $p(Y(0) \mid S = s_{ij}, Z = z) \neq p(Y(0) \mid S = s_{km}, Z = z)$. However, this implies that the probabilities p_{00}, p_{01}, and $p_{1\bullet}$ must be fine-tuned in the sense that they produce perfect cancellations of terms in Eq. (S5) such that the equality holds. This establishes claim (ii).

Throughout, the same logic applies for $a = 1$, establishing the desired results. □

Appendix S3: Numerical example of identification in class $\mathcal{N}$ but not $\mathcal{F}$

Theorem 1 and Lemma 1 together imply that if (C1) and (C2) hold but $R \in De(A)$, then there exists some model $M \in \{\mathcal{F} \setminus \mathcal{N}\}$ such that δ_R is not identified. We give a numerical example for a graph in which (C1) and (C2) hold (guaranteeing that δ_R is identified for the

class $\mathcal{N}$), yet in which δ_R is not identified for the broader class $\mathcal{F}$. Consider the structure $A \to R$ with Y d-separated from both A and R, such that:

$$A = \epsilon_A \text{ where } \epsilon_A \sim \text{Bern}(0.3)$$

$$Y(a) = \epsilon_Y \text{ where } \epsilon_Y \sim \text{Bern}(0.4)$$

$$R(a) = \max\{a, \epsilon_R\} \text{ where } \epsilon_R = \epsilon_Y$$

This distribution M is a member of $\mathcal{F}$ but not of $\mathcal{N}$ because $\epsilon_R \not\perp\!\!\!\perp \epsilon_Y$. Noting that $Y(a) = Y$ since $Y \notin De(A)$ and that $P(R = 1) = 0.58$,[e] we have:

$$p(Y(1) = 1 \mid R = 1) = \frac{p(R = 1 \mid Y = 1)P(Y = 1)}{P(R = 1)} = \frac{0.4}{0.58} \approx 0.690 \tag{S6}$$

On the other hand, since $Y \perp\!\!\!\perp A$ marginally:

$$\begin{aligned} p(Y = 1 \mid A = 1, R = 1) &= \frac{P(R = 1 \mid Y = 1, A = 1)P(Y = 1)}{\sum_{y \in \{0,1\}} P(R = 1 \mid Y = y, A = 1)P(Y = y)} \\ &= \frac{P(Y = 1)}{\sum_{y \in \{0,1\}} P(Y = y)} \\ &= 0.4 \end{aligned}$$

Since $p(Y(1) = 1 \mid R = 1) \neq p(Y = 1 \mid A = 1, R = 1)$ for this distribution $M \in \mathcal{F}$, δ_R is not R-identified.

[e] $0.58 = 0.3 * 1 + 0.7 * 0.4 * 1 + 0.7 * 0.6 * 0$

References

[1] Daniel Malinsky, Ilya Shpitser, and Thomas Richardson. "A potential outcomes calculus for identifying conditional path-specific effects." *The 22nd International Conference on Artificial Intelligence and Statistics*. PMLR. 2019, pp. 3080–3088.

[2] Maya B Mathur, Ilya Shpitser, and Tyler J VanderWeele. "A common-cause principle for eliminating selection bias in causal estimands through covariate adjustment" (2023). Preprint retrieved from `https://osf.io/ths4e/` (version 3).

[3] Thomas S Richardson and James M Robins. "Single world intervention graphs (SWIGs): A unification of the counterfactual and graphical approaches to causality." *Center for the Statistics and the Social Sciences, University of Washington Series. Working Paper* 128.30 (2013), p. 2013.

[4] Judea Pearl. *Causality*. Cambridge University Press, 2009.

[5] Constantine E Frangakis and Donald B Rubin. "Principal stratification in causal inference." *Biometrics* 58.1 (2002), pp. 21–29.
